# Preliminary Validation of Direct Detection of Foot-And-Mouth Disease Virus within Clinical Samples Using Reverse Transcription Loop-Mediated Isothermal Amplification Coupled with a Simple Lateral Flow Device for Detection

**DOI:** 10.1371/journal.pone.0105630

**Published:** 2014-08-28

**Authors:** Ryan A. Waters, Veronica L. Fowler, Bryony Armson, Noel Nelson, John Gloster, David J. Paton, Donald P. King

**Affiliations:** 1 Livestock Viral Disease Program, The Pirbright Institute, Pirbright, Guildford, Surrey, United Kingdom; 2 Atmospheric Dispersion and Air Quality, The Meteorological Office, Exeter, United Kingdom; Centro de Biología Molecular Severo Ochoa (CSIC-UAM), Spain

## Abstract

Rapid, field-based diagnostic assays are desirable tools for the control of foot-and-mouth disease (FMD). Current approaches involve either; 1) Detection of FMD virus (FMDV) with immuochromatographic antigen lateral flow devices (LFD), which have relatively low analytical sensitivity, or 2) portable RT-qPCR that has high analytical sensitivity but is expensive. Loop-mediated isothermal amplification (LAMP) may provide a platform upon which to develop field based assays without these drawbacks. The objective of this study was to modify an FMDV-specific reverse transcription–LAMP (RT-LAMP) assay to enable detection of dual-labelled LAMP products with an LFD, and to evaluate simple sample processing protocols without nucleic acid extraction. The limit of detection of this assay was demonstrated to be equivalent to that of a laboratory based real-time RT-qPCR assay and to have a 10,000 fold higher analytical sensitivity than the FMDV-specific antigen LFD currently used in the field. Importantly, this study demonstrated that FMDV RNA could be detected from epithelial suspensions without the need for prior RNA extraction, utilising a rudimentary heat source for amplification. Once optimised, this RT-LAMP-LFD protocol was able to detect multiple serotypes from field epithelial samples, in addition to detecting FMDV in the air surrounding infected cattle, pigs and sheep, including pre-clinical detection. This study describes the development and evaluation of an assay format, which may be used as a future basis for rapid and low cost detection of FMDV. In addition it provides providing “proof of concept” for the future use of LAMP assays to tackle other challenging diagnostic scenarios encompassing veterinary and human health.

## Introduction

Incursions of foot-and-mouth disease (FMD) into countries or zones with FMD-free status has devastating impacts. Although the clinical manifestations of FMD can be severe, the primary impact of an outbreak is that there is an immediate restriction on international trade of animals and animal products. An outbreak therefore has a huge economic consequences on the country, a result of both direct and indirect monetary losses. For example the 2001 UK FMDV outbreak was estimated to have cost over £8 billion [1] with a resultant drop of national GDP in that year of 0.2%. As a result of the economic impact in the event of an incursion of FMD, the primary goal is to return to FMD free status as quickly as possible after the first case is identified to minimise these impacts upon national livestock industries.

Rapid and accurate detection of FMDV is one of the first steps in the control pathway and therefore central to minimising spread of the disease. Samples from suspected outbreaks can be tested for the presence of virus by (i) RT-qPCR [2], (ii) FMDV antigen ELISA [3], and (iii) virus isolation [4]. Although some of these tests are rapid, they all rely upon the transport of samples from suspect cases to centralised laboratories which can add a significant time delay from sample collection to arrival at the laboratory. This is especially relevant if the distances involved are large. In addition to rapidity, the ability to detect pre-clinical infection is also desirable to maximise the impact of subsequent control measures. Due to the required rapidity of responses to the UK 2001 outbreak demanded at the time, many premises were slaughtered without confirmatory laboratory diagnosis. Retrospective analysis of samples collected on these premises could not find any evidence of FMD circulation on 23% of the farms designated as infected [5]. Moreover, the widespread dissemination of FMD in the UK in 2001 has been largely attributed to the silent spread in sheep [6], which would render field based assays reliant upon clinical signs useless in this species as sheep are well documented to often show minimal to no clinical signs associated with FMDV infection [7].

Two of the main reports published after the UK 2001 outbreak recommended the development of rapid field based diagnostics [8], [9]. The benefits of such assays are not only restricted to previously free countries either. Many endemic countries in the developing world have relatively poor infrastructure and under-funded laboratory facilities. Accurate, rapid, and cheap diagnostic tests which are able to be implemented on farm would enable the majority of clinical cases suspected to be FMD, to be confirmed and reported to a central facility with relative ease and confidence. Accumulation of this outbreak data has been recognised as an essential first step in both longer term progressive control of the disease and also of an early warning system for nascent outbreaks of FMD within an endemic country [10].

To date, two main technologies exploiting either antigen or nucleic acid detection methods have been targeted for incorporation into portable diagnostic platforms [11], [12]. An immunochromatographic lateral flow device (LFD) to directly detect viral antigen, with equivalent diagnostic sensitivity to the laboratory based antigen ELISA, has been developed for use in the field as a pen side test [13]. This assay is extremely portable and easy to use, giving a result in as little as 10 minutes. However, only a limited number of sample types can be tested using the LFD which must contain large amounts of FMD viral antigen in order to generate a positive result. These factors restrict the usefulness of this test to the acute clinical phase of FMD where diseased epithelium is collectable (up to 3–4 days after the onset of lesions) and contains large amounts of intact FMDV antigen. Beyond this time, either no epithelium is available to process, or viral antigen has degraded to a level no longer detectable by the test (Unpublished field observations). As described previously, some animals may not show obvious clinical signs during the entirety of infection with FMDV, making the use of this LFD redundant in these situations and by extension during the incubation period.

Detection of viral nucleic acid using real time RT-qPCR is recognised as having a much higher analytical sensitivity than antibody-based assays for the detection of FMDV [14]. Because very small quantities of viral RNA are able to be detected with the real time RT-qPCR, the diagnostic window applicable to this test is much wider than for the LFD, including the ability to detect FMDV RNA during the pre-clinical phase of infection. This, therefore, results in it having a much higher diagnostic sensitivity than the LFD. Indeed pre-clinical detection of FMDV in animals was demonstrated in the field during the 2007 FMD outbreak in the UK using RT-qPCR [15].

FMDV-specific real-time RT-qPCR assay chemistry has been transferred into portable PCR platforms and evaluated for diagnostic use [16]–[19]. These assays maintain high analytical sensitivity equivalent to that of a laboratory based test [19], and also a higher diagnostic sensitivity than the LFD. The hardware required for these assays is, however, relatively expensive since it uses a complex protocol for nucleic acid extraction and also needs precision temperature control for the amplification step. Furthermore, decontamination of such complex instrumentation is difficult. In light of these drawbacks, it is likely that such an assay would not be used as an on-farm pen side test, but would rather be positioned in regional veterinary laboratories close to (or within) the outbreak foci [12]. Given these limitations, a more cost-effective format for molecular diagnostics of FMD on farm, whilst maintaining the highest analytical sensitivity, is required.

In 2000, a novel nucleic acid amplification chemistry was developed called Loop-mediated isothermal AMPlification (LAMP). LAMP amplifies a nucleic acid target at a single constant temperature, relying on the strand displacement activity of a *Bst* DNA polymerase enzyme, negating the need for thermal cycling [20]. LAMP methods have been shown to have similar analytical sensitivity to RT-qPCR or PCR and do not require an expensive thermal cycler for the amplification of target sequences. LAMP assays have now been developed for the detection of multiple infectious disease organisms including bacteria [21], protozoa [22] and viruses [23]. An FMDV-specific RT-LAMP assay has previously been developed, which was able to rapidly detect FMDV RNA extracted from lesion material with equivalent analytical sensitivity to the laboratory based RT-qPCR [24]. Detection of RT-LAMP products was achieved either by analysis using agarose gel electrophoresis or utilisation of an intercalating dye combined with real-time PCR machines, both of which were effective but not practical to use as a pen side method. Turbidity [25] and colour change detection formats have been reported as more portable and simple direct visual methods for the detection of LAMP products for other pathogens, but are sometimes difficult to interpret.

Detection of LAMP products has been done by using LFD end point detection techniques [26]–[29] but have drawbacks of relatively fastidious conditions. More recently, detection of dual labelled LAMP products has been demonstrated using bespoke low cost LFDs operating on a much simpler antigen-antibody interaction. This simple approach enabled simple LAMP assay detection of African Swine Fever Virus (ASFV) [30]. Unequivocal positive or negative results that can be interpreted by a non-specialist can thus be obtained at low cost.

Here, we describe the modification of a previously reported FMDV-specific RT-LAMP assay, to allow detection of dual labelled LAMP products with a commercially available LFD. Furthermore, this study also investigated the practicality of simple methods that could be applied for sample preparation in the field.

## Results

### Development and validation of RT-LAMP-LFD assay using RNA standards

**Figure 1:**
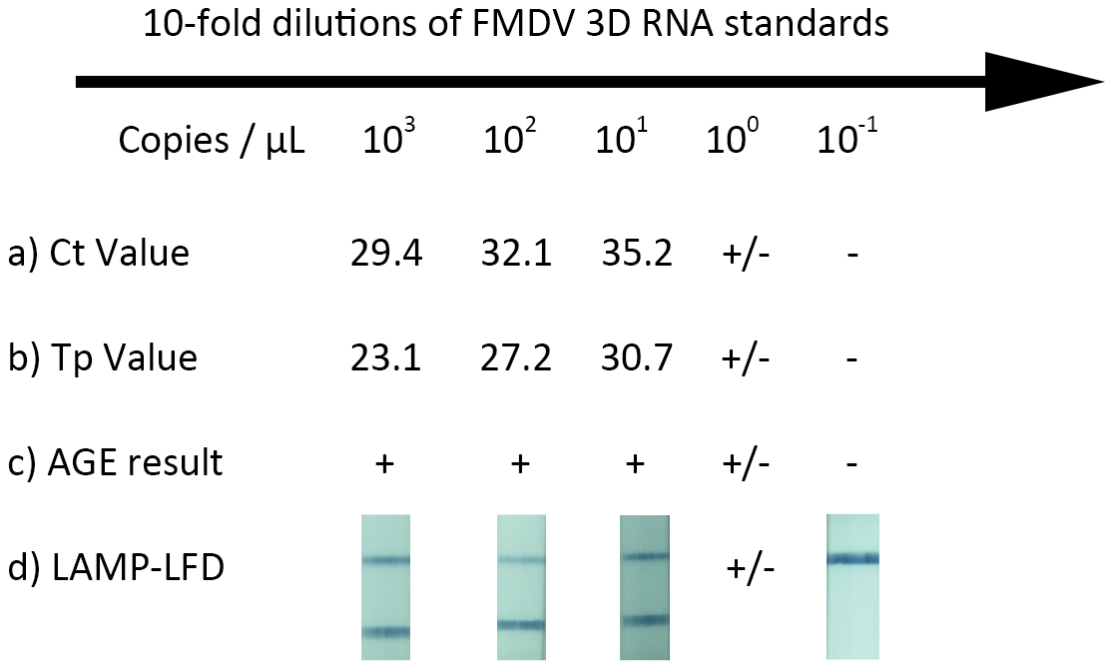
Limit of detection between the RT-qPCR (Ct value), RT-LAMP (Tp value) and RT-LAMP LFD assays. (a) RT-qPCR amplification Ct values corresponding to each of the 10-fold dilutions of RNA standards; (b) RT-LAMP amplification result with Tp values given for each RNA copy number; (c) AGE analysis of RT-LAMP-LFD reaction, spanning the same RNA standards as in (a) and (b); (d) The RT-LAMP-LFD reactions from (c) applied to the LFD device. +/− indicates that out of 4 identical replicates of a given RNA concentration applied to a specific assay (a–d), there were a mixture of positive and negative results.

The limit of detection for the one step RT-qPCR assay was established using FMDV RNA standards (O/UKG/34/2001). RNA standards of concentrations from 10^7^ copies/µl to 10^1^ copies/µl (inclusive) were consistently detected (Figure 1a). At higher dilutions, 50% of reactions containing 10^0^ copies/µl RNA standards were detected, while all of the dilutions at 10^−1^ copies/µl were negative. In light of this data, four dilutions spanning consistently positive (10^2^ and 10^1^), intermediate (10^0^) and negative (10^−1^) by RT-qPCR were taken forward to subsequently evaluate the performance of the RT-LAMP assay.

Preliminary experiments examined the performance of the already existing RT-LAMP chemistry compared to RT-qPCR. In these experiments where the RT-LAMP was performed using a real-time PCR machine, the limit of detection between the RT-LAMP and RT-qPCR assay were equivalent (Figure 1b). Furthermore, agarose gel electrophoresis (AGE) end-point examination of RT-LAMP products agreed with these results generated on a real-time PCR machine with PicoGreen as an indicator of amplified LAMP products (Figure 1c). However, in these experiments it was not possible to evaluate the RT-LAMP-LFD assay using the real-time PCR machine due to interference of the fluorescein (flc) labelled primers with PicoGreen detection. Therefore these results using the labelled primers were visualized using LFD’s and AGE. Biotin and fluorescein labelling of the oligos used in the RT-LAMP assay (termed RT-LAMP-LFD) had no effect on the limit of detection of the assay when the reactions were analysed by both AGE and the LFD (Figure 1d). Of note was the fact that the intensity (and thus quantity) of the positive bands on AGE from the RT-LAMP-LFD were all similar, and did not reduce in intensity as the limit of detection was approached, reflecting a clear positive-negative distinction. Importantly, when these reaction products were applied to the LFD, negative and positive results completely matched those revealed by AGE, with equivalent intensity of positive bands being noted (Figure 1d). The positive bands, when present, were evident within one minute of application to the LFD.

### Determination of the optimum temperature range for the RT-LAMP and RT-LAMP-LFD assays

The impact of varying the incubation temperature from 55–75°C upon assay sensitivity was assessed for the RT-LAMP and RT-LAMP-LFD assays. Each assay was performed at a range of temperatures and amplification products detected using AGE. The RT-LAMP reaction maintained a limit of detection comparable to that of the RT-qPCR assay when incubated at temperatures between 55.3 and 63.2°C (Table 1). Amplification appeared to be non-specific below 55.3°C, while at 71°C and higher, no amplification was observed with any of the RNA samples tested. The RT-LAMP-LFD maintained the same limit of detection as the RT-qPCR between 55.3 to 63.2°C, a slightly narrower window than the RT-LAMP assay (Table 1). At 65.9°C and 68.5°C, the limit of detection was reduced 10 fold to 10^1^ copies RNA, with no amplification observed at 71.0°C and above. The reaction products from the RT-LAMP-LFD were also tested using the LFD: all products that yielded positive results for AGE were also positive using the LFD, while all reactions negative using AGE were also negative when using a LFD. All positive LFD results could be seen within one minute after application of the amplification product to the device.

**Table 1:** The effect of isothermal temperature on the end point limit of detection of both the RT-LAMP (un-labelled internal primers) and RT-LAMP-LFD (labelled internal primers) reaction.

		RT-LAMP - temperature/°C											
RNA copies/µl	Mean Ct Value	55	55.3	56.5	58.3	60.6	63.2	65.9	68.5	71	73.1	74.6	75.4
10^2^	32.3	+	+	+	+	+	+	+	+	−	−	−	−
10^1^	35.5	+	+	+	+	+	+	+	−	−	−	−	−
10^0^	+/−	+	+	+/−	+/−	+/−	+/−	−	+/−	−	−	−	−
10^−1^	–	+	−	−	−	−	−	−	−	−	−	−	−
Neg control	–	+	−	−	−	−	−	−	−	−	−	−	−
		**RT-LAMP-LFD - temperature/°C**											
**RNA copies/µl**	**Mean Ct Value**	**55**	**55.3**	**56.5**	**58.3**	**60.6**	**63.2**	**65.9**	**68.5**	**71**	**73.1**	**74.6**	**75.4**
10^2^	32.3	+	+	+	+	+	+	+	+	−	−	−	−
10^1^	35.5	+	+	+	+	+	+	−	+	−	−	−	−
10^0^	+/−	+/−	+/−	+/−	+/−	+	+	−	−	−	−	−	−
10^−1^	−	−	−	−	−	−	−	−	−	−	−	−	−
Neg control	−	−	−	−	−	−	−	−	−	−	−	−	−

RNA standards were used to define the limit of detection, with the RT-qPCR Ct values being displayed for each RNA dilution. The RNA standards span the limit of detection of the RT-qPCR assay.+indicates reaction was positive by gel electrophoresis and PicoGreen fluorescence analysis (for un-labelled primers) or by LFD analysis (for labelled primers). +/− indicates variable positivity amongst quadruplicates analysed for a given RNA copy number.

### Detection of FMDV RNA by RT-LAMP-LFD in a simple format

**Figure 2:**
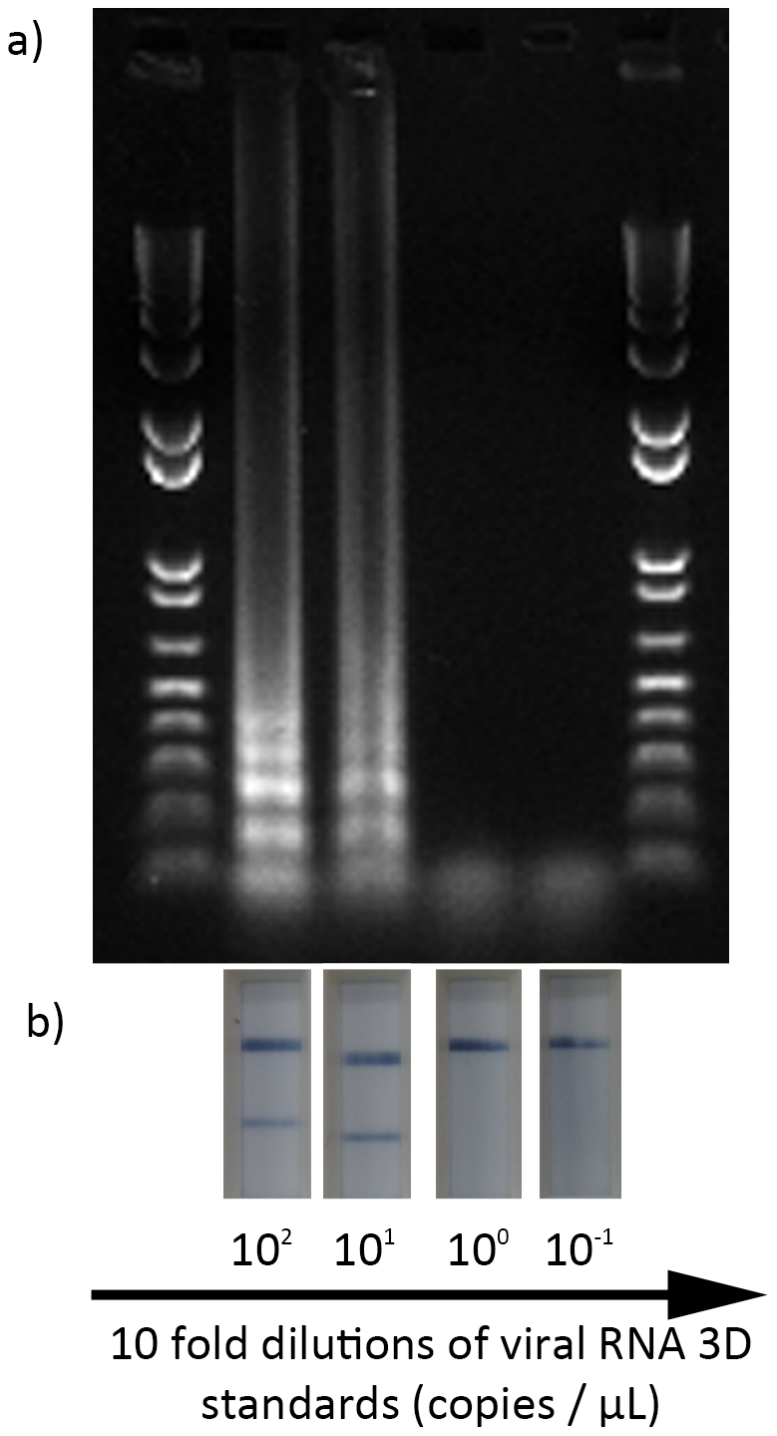
RT-LAMP-LFD reactions utilising a simple desk top water bath as the isothermal heat source. (a) RT-LAMP-LFD products analysed using AGE; (b) The RT-LAMP-LFD products from (a) applied to the LFD.

Based on the results above, a water bath set to 60°C was able to act as the heat source for the RT-LAMP-LFD reactions (Figure 2). After an incubation period of 60 minutes, the dual-labelled reaction products were analysed by LFD and results were confirmed using AGE. Using these conditions, the RT-LAMP-LFD assay consistently detected the RNA standards at 10^1^ copies/µl (Figure 2). Samples run in parallel on an electronic heat block gave identical results (data not shown).

**Figure 3:**
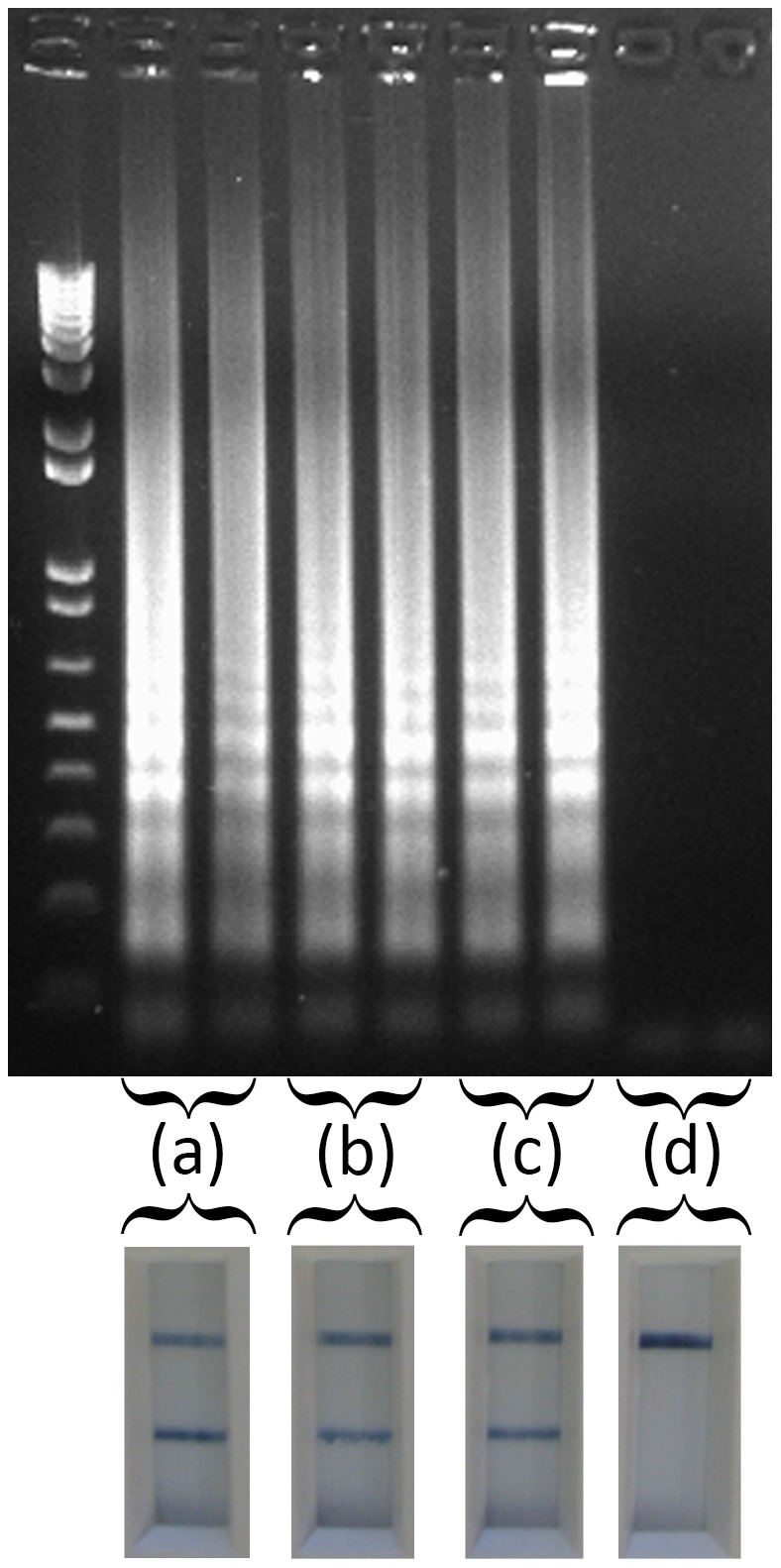
Titration of epithelial suspensions and subsequent analysis with the RT-LAMP-LFD assay. A 10% epithelial homogenate (w/v) containing 10^6^ TCID_50_/mL FMDV was diluted in nuclease free water as described below. These dilutions were each assayed in duplicate with the RT-LAMP-LFD protocol, and analysed with AGE and on an LFD. (a) Application of 5 uL of the “neat” 10% epithelial homogenate added directly to the RT-LAMP-LFD reaction, (b) 1 in 3 dilution, and (c) 1 in 5 dilution, (d) 5 ul of nuclease free water added to the reaction mixture.

Due to the reported insensitivity of the LAMP chemistry to inhibiting factors that might be present in clinical samples [31], the ability to perform the RT-LAMP-LFD directly on epithelial samples containing FMDV was evaluated. FMDV spiked 10% epithelial suspension, and non-spiked (negative) 10% epithelial solutions were each used neat or pre-diluted 1 in 3 and 1 in 5 in nuclease free water. Five µl of each dilution of the spiked and non-spiked suspensions were analysed using the RT-LAMP-LFD assay using a water bath and an incubation step of 60°C for 60 minutes. Positive RT-LAMP-LFD results were obtained for all dilutions of the positive epithelium suspension (Figure 3). Intermittent DNA AGE patterns, not consistent with RT-LAMP products were detected in neat negative epithelial suspensions and those diluted 1 in 3 indicative of non-specific reactions. Furthermore, these reactions, when run on the LFD, always generated negative results. All negative epithelial suspensions analysed at a 1 in 5 dilution, gave no AGE bands and were also negative after LFD interrogation. As a result, all 10% epithelial suspensions were pre-diluted 1 in 5 with nuclease free water before all subsequent RT-LAMP-LFD analyses.

### Limit of detection of the pen-side RT-LAMP-LFD assay

**Figure 4:**
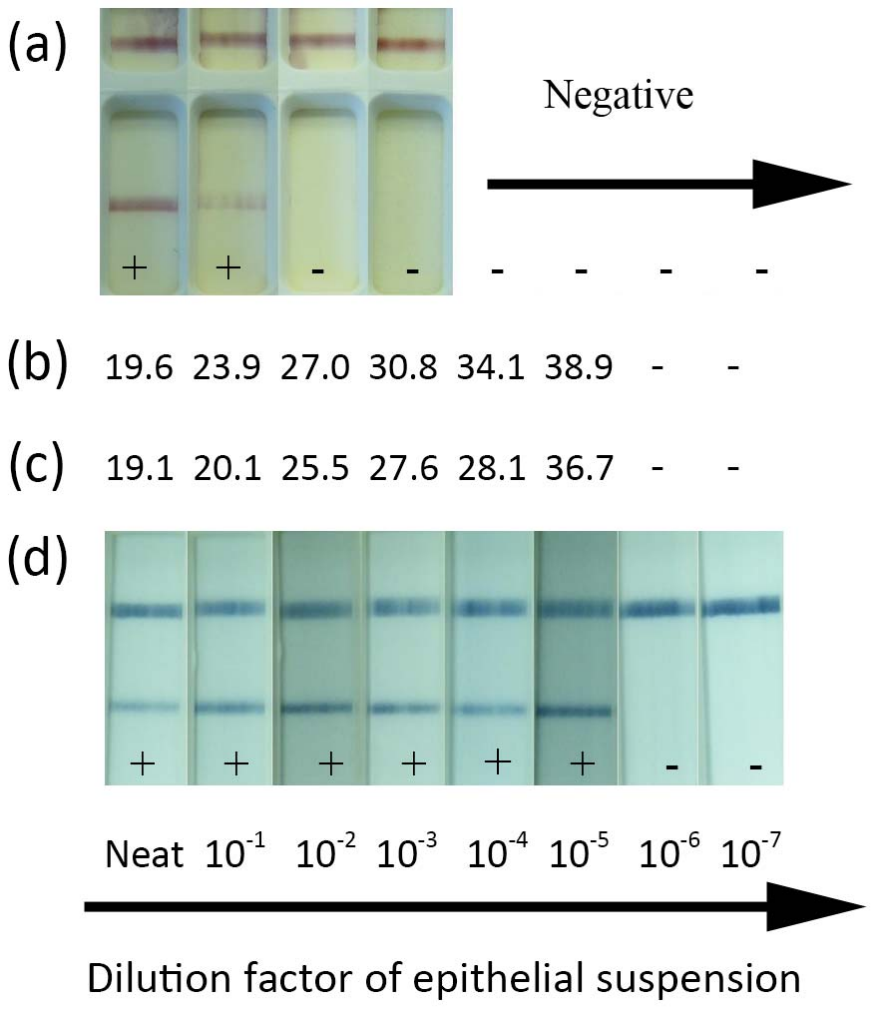
Comparative limit of detection between Svanova LFD, RT-qPCR, RT-LAMP-LFD and the RT-LAMP assays. 10 fold dilutions of FMDV containing epithelial suspensions were analysed using the Svanova LFD device (a) by applying the suspensions directly to the device. RNA was extracted from each suspension and subsequently analysed using either the RT-qPCR (giving a Ct value) (b) the RT-LAMP assay read using PicoGreen fluorescence(giving a Tp value) (c) or by mixing each 10 fold dilution 1∶5 with nuclease free water, and directly adding this to the RT-LAMP-LFD assay for subsequent detection with the LFD (d).

The performance of the optimised RT-LAMP-LFD assay was compared against the RT-qPCR, as well as the FMDV antigen LFD (SVANODIP FMDV-Ag, Svanova) currently marketed for field diagnosis of FMD [13]. Using a decimal dilution series of FMDV spiked 10% epithelial suspensions as the starting sample, the ability of these different assays to detect FMDV was compared. The antigen LFD gave positive results when neat and 10^−1^ dilutions of epithelium were analysed; however, all other dilutions were negative (Figure 4a). RNA robotically extracted from the spiked epithelial suspension dilution series gave positive signals from the neat to the 10^−5^ dilutions of the epithelial suspensions (Figure 4b). The optimised RT-LAMP assay had an equivalent limit of detection as the RT-qPCR assay on detecting this extracted RNA, when analysed on the real-time PCR machine and Tp values examined (Figure 4c). Independent processing of this dilution series was undertaken using the optimised RT-LAMP-LFD assay including pre dilution of each epithelial suspension 1 in 5 with nuclease-free water, isothermal amplification using a water bath and detection the RT-LAMP products with the LFD (Figure 4d). RT-LAMP-LFD was found to generate concordant results to those obtained using automated real-time RT-qPCR, with clear detection of FMDV RNA within the dilution range of neat to 10^−5^.

### Direct detection of FMDV from field samples

Epithelial suspensions representing four serotypes (A, Asia 1, SAT1 and SAT2) were analysed using the direct RT-LAMP-LFD protocol. Following the 60 minute water bath incubation at 60°C, the dual labelled reaction products were visualised by LFD and results confirmed by AGE (Table 2c). For comparison total nucleic acid was extracted from the original epithelial suspensions using a robot (MagNA pure LC, Roche). This RNA (5 µl) was then assayed in parallel by real-time PCR and RT-LAMP using a PCR machine (Stratagene) and visualised by fluorescence. In addition the epithelial suspensions were applied directly to the FMDV antigen LFD (Table 2d). FMDV was detected by all assays from all four serotypes assessed (Table 2), with the negative controls all negative.

**Table 2:** Comparison between the RT-qPCR, RT-LAMP and RT-LAMP-LFD assays from field epithelial suspensions.

	Epithelial suspension	Asia 1		A			SAT 1	SAT 2	
		TUR 2/2013	PAK 21/2012	IRN 24/2012	TUR 7/2013	TUR 4/2013	TAN 50/2012	TAN 14/2012	BOT 15/2012
**(a) RT-qPCR (Ct)**	No Ct	12.52	12.54	13.93	12.6	19.33	25.65	14.75	18.79
**(b) RT-LAMP (Tp)**	No Ct	26.41	20.34	23.21	17.74	30.63	47.82	21.44	27.88
**(c) AGE**	−	+	+	+	+	+	+	+	+
**(d) Svanova LFD**	**−**	+	+	+	+	+	+	+	+
**(e) RT-LAMP-LFD**	**−**	+	+	+	+	+	+	+	+

(a) RT-qPCR amplification Ct values corresponding to each of the 1∶5 dilutions of FMDV epithelial suspensions from a range of serotypes; (b) RT-LAMP amplification result with Tp values given for each epithelial suspension; (c) AGE analysis of LAMP-LFD reaction, spanning the same epithelial suspensions as in (a) and (b); (d) epithelial suspension analysed by Svanova antigen LFD; (e) The RT-LAMP-LFD reactions from (c) applied to the LFD device.

### Direct detection of FMDV RNA in air samples

Following successful evaluation of the finalized RT-LAMP-LFD assay on field epithelial samples, air samples collected with one of two portable air samplers during experimental FMDV transmission experiments were also analysed. Parallel testing of these samples was carried out using automated real-time RT-qPCR and the results are summarised in Table 3. The real-time RT-qPCR assay detected viral RNA, collected using a Biocapture 650 over a period of 30 minutes, from the air of a box containing five sheep at both one and two days post infection (dpi). FMDV RNA could also be detected using the real-time RT-qPCR assay in air samples collected in the same way in a box housing cattle at two dpi. Air samples from a box containing five pigs were positive for viral RNA when analysed using the real-time RT-qPCR assay on all days (one, two, three and four dpi). These same samples were analysed using the RT-LAMP-LFD assay, by adding 5 ul of the aqueous sample directly into the RT-LAMP-LFD reaction mix. Apart from an air sample collected from the sheep box (at two dpi), there was complete concordance between the results generated by the RT-LAMP-LFD and the real-time RT-qPCR (Table 3). The aforementioned discrepant result had the lowest recorded positive signal for any of the real-time RT-qPCR positive samples (Table 3). These results were irrespective of whether the air samples had been collected with either the Biobadge 100 (3 hour run time or 20 hour run time) or Biocapture 650. Furthermore, positive RT-LAMP-LFD results were generated for pigs (at one dpi) that were not showing overt clinical signs.

**Table 3:** RT-LAMP-LFD Detection of FMDV in air samples collected with 2 different portable air samplers (Biobadge 100 and Biocapture 650).

			Sample collection/days post infection				
Species infected	Air sampling instrument used	Assay utilised	0	1	2	3	4
Pig	Biobadge (short Run ∼3 hrs)	RT-qPCR (Ct Value)	No Ct	25.8	23.52	30.07	27.66
		LAMP-LFD	−	+	+	+	+
	Biobadge (long run ∼20 hrs)	RT-qPCR (Ct Value)	No Ct	20.74	23.51	24.02	28.19
		LAMP-LFD	−	+	+	+	+
	Biocapture (∼30 mins)	RT-qPCR (Ct Value)	No Ct	22.41	23.49	23.68	29.74
		LAMP-LFD	−	+	+	+	+
Cattle	Biocapture (∼30 mins)	RT-qPCR (Ct Value)	No Ct	No Ct	33.84	No Ct	No Ct
		LAMP-LFD	−	−	+	−	−
Sheep	Biocapture (∼30 mins)	RT-qPCR (Ct Value)	No Ct	35.78	37.31	No Ct	No Ct
		LAMP-LFD	−	+	−	−	−

RNA was extracted and analysed using the RT-qPCR assay, with Ct values being displayed.

Raw air samples were applied to the RT-LAMP-LFD assay, and analysed using the LFD. Air samples were collected from day 0 (day of inoculation), every day, until day 4. Species sampled were pigs, cattle and sheep.

## Discussion

A range of LAMP and RT-LAMP assays have been developed to detect nucleic acids from a wide variety of different pathogens that impact upon veterinary and human health. The development of portable hardware and companion protocols to enable LAMP assays to be deployed into the field (or clinic), however, has taken lower priority to date. LAMP has a number of characteristics that are particularly suitable for incorporation into a simple assay format. These include: synthesis of large amounts of DNA which can be readily detected using agarose-gel electrophoresis, or using a fluorescence plate reader in combination with fluorescent intercalating dyes (such as a real-time PCR machine). These approaches are not currently suitable as the basis for a simple test that could be used in the field. Alternative methods that are being considered for simple detection of LAMP include the use of turbidity equipment to monitor the accumulation of insoluble magnesium pyrophosphate that is generated as a white precipitated bi-product of the LAMP amplification [25], or the use of dyes such as hydroxynaphthol blue that respond to changes in cation (Mg^2+^) concentration associated with LAMP amplification [32]. However, these indirect measurements of LAMP products are prone to generating false negative results and are not currently robust enough for field use. Furthermore, without specific hardware, these forms of detection fail to generate a binary positive/negative result.

In this study, we have modified an existing FMDV RT-LAMP assay in such a way that the entire process can be performed without the use of expensive equipment. The labelling of the primers to allow direct detection of the RT-LAMP products had no effect on assay limit of detection. It should be pointed out, however, that the primer pair which were conjugated to Flc/Biotin to enable successful LFD detection of products in the study described here were the forward and backward internal primers (FIP/BIP). Preliminary experiments were undertaken using a different Flc/Biotin labelled primer pair, namely the forward and reverse loop primers (Floop/Bloop). This approach was initially tried due to success utilising this approach in development of an ASFV LAMP-LFD assay [30]. Whilst successful for the ASFV-LAMP assay, it failed when applied to the FMDV-LAMP assay, hence the ultimate switch to using the labelled FIP/BIP approach. This disparity most likely reflects differences in the ratios to which the different primer sets are incorporated into the complex array of RT-LAMP products, which itself will be related to the identity of the specific primer sequences. This is an important consideration whilst adapting LAMP assays to this LFD format. The limit of detection of the direct simplified assay was such that it was equivalent to that of a validated real-time RT-qPCR assay used for diagnosis in National and International FMD Reference laboratories. The similar intensities of positive bands on both AGE analysed RT-LAMP and RT-LAMP-LFD products illustrated that when amplification does occur, a similar amount of end product is generated, regardless of the input copy number. Importantly, because this polarized digital nature of product quantity was mirrored by the LFD analysis of RT-LAMP-LFD, it meant that interpretation of these devices was straight forward and less open to subjective interpretation, even when samples at the limit of detection were examined. These results are in contrast to assay results generated using an FMDV antigen LFD (SVANODIP FMDV-Ag, Svanova) where the intensity of the band was proportional to the amount of viral antigen in the sample and results can be difficult to interpret when tested samples comprise FMDV at, or near to, the limit of detection. The implication of this is that the RT-LAMP-LFD assay most likely has a wider diagnostic window than the antigen LFD, being able to detect viral RNA for much longer time points post infection than the antigen LFD. This hypothesis should be investigated by testing a multitude of sample types and multiple time points post infection using both the antigen LFD and the RT-LAMP-LFD.

We observed 100% concordance between the results generated using the LFD and AGE methods for both positive and negative samples, demonstrating that a LFD approach to the analysis of RT-LAMP reactions is both sensitive and robust.

In addition to a simple and inexpensive method to detect RT-LAMP reaction products, this study has also demonstrated that cDNA amplification by RT-LAMP is not reliant upon a stringent isothermal incubation step, since the analytical sensitivities of the RT-LAMP assays were maintained over a wide range of temperatures (8.2°C for the unlabelled and labelled RT-LAMP assays). This allows RT-LAMP amplification to be performed in a water bath set to 60°C as the heat source. The successful amplification and detection of the RNA targets using these conditions, whilst having no impact upon the limit of detection of the assay, provides a practical demonstration that a tightly controlled temperature afforded by a thermal cycler is not necessary for the RT-LAMP-LFD assay. These findings are important since, when compared to the requirement for precise thermal incubation steps, functionality of the assay over a wide temperature range has the potential to reduce the cost of a portable heat source that might be used as the basis for an assay deployed into the field. Furthermore, the ability for amplification to be efficient at multiple temperatures may allow a wider number of heat sources, for example the use of exothermic reactions.

The production of template nucleic acid free of tissue-derived PCR inhibitory factors is an important consideration when developing molecular assays for the detection pathogens in the field. This aspect has previously been recognised as a limitation to field deployment of molecular methods for pathogen detection [18]. Unlike PCR, previous studies have indicated that LAMP is not inhibited to the same extent by contaminants that might be carried over from the sample [33] allowing amplification even with relatively crude extraction procedures [34]. Our study provides evidence that simply diluting the raw epithelial suspension with nuclease-free water is sufficient for the efficient amplification of FMDV using RT-LAMP-LFD. Similarly, air sample fluid from a portable air sampler can be added directly to the RT-LAMP-LFD reaction mix without compromising analytical sensitivity when compared to a diagnostic real-time RT-qPCR assay. Dispensing with complex RNA extraction methods is a major step forward in portable nucleic acid detection platforms, as all that is required is a heat source, an LFD and the RT-LAMP-LFD reaction master mix.

This study compares the performance of front line tests that can be used for FMD diagnosis including an antigen LFD (SVANODIP FMDV-Ag, Svanova) and real-time RT-qPCR with the RT- LAMP-LFD assay. Our results show that the simplified RT-LAMP-LFD assay utilising a raw sample is 10^4^ times more sensitive at detecting the presence of FMDV compared with the FMDV-specific antigen LFD (SVANODIP FMDV-Ag, Svanova). Furthermore, when compared to current “portable” real time RT-qPCR assays/equipment, there is a much wider scope for higher throughput of the LAMP-LFD assay described here, due to robust chemistry conditions, no need for RNA extraction, and simple detection methodology. FMDV RNA could be detected using the finalised RT-LAMP-LFD protocol, from field samples containing four currently circulating serotypes of FMDV from around the world. This demonstrates a potential geographical and serotypic robustness of this assay and its use as a diagnostic tool in multiple endemic countries. Furthermore, these data suggest that the RT-LAMP-LFD assay may also have a longer diagnostic temporal window in which FMDV can be detected, compared to the antigen LFD. In part, this conclusion is supported by the ability of the RT-LAMP-LFD assay to detect low amounts of FMDV in air samples collected with portable samplers, including air samples from groups of infected pigs in the pre-clinical stage of disease. In addition to the obvious value of this assay in the urgent detection of FMD cases in normally FMD free countries, field detection of FMD will also assist the diagnosis of the disease in developing countries that have limited laboratory capability and/or difficult transport links that do not facilitate rapid submission of clinical samples.

In conclusion, we present the development and evaluation of a robust FMDV diagnostic assay using RT-LAMP chemistry, the results of which can be observed using a simple LFD. The unequivocal nature of the positive and negative readings is important for field based formats where subjective interpretation of test results is undesirable. There is no requirement for prior RNA extraction, thus allowing the simple addition of raw epithelial homogenates and raw air samples containing FMDV to the assay directly, whilst maintaining a similar limit of detection as the “gold standard” real-time RT-qPCR assay after extraction of RNA. The assay also has a wide operating temperature, negating the need for strict temperature regulation. These are ideal characteristics for a pen-side assay for FMDV. Further work is now required to formulate this assay into a kit format suitable for routine use, in addition to continuing validation and assessment of further sample types. The findings presented here for the detection of FMDV may also impact upon diagnostic scenarios for other veterinary and human diseases where the rapid and simple detection of nucleic acid targets are warranted.

## Methods

### Ethics statement

All animal samples utilised in this paper were archival samples from previous studies approved by The Pirbright Institute ethical review committee under the auspices of the Animal Scientific Procedures Act (ASPA) 1986 (as amended).

### Epithelial suspensions and air samples

#### Spiked epithelial suspensions

A 10% tongue epithelial homogenate in sample preparation buffer (SVANODIP, Svanova) was prepared from uninfected sheep epithelium. To prepare the spiked epithelium suspension, the 10% tongue suspension was spiked 1∶100 with 10^8^ TCID_50_/ml of FMDV UKG 34/2001, to create a “neat” stock of 10% tongue homogenate containing a titre of 10^6^ TCID_50_/ml FMDV.

#### Field sample epithelial suspensions

Epithelial suspensions prepared from samples submitted to The World Reference Laboratory for FMD (Pirbright, UK), representing serotypes A (TUR 2/2013; PAK 21/2012), Asia 1 (IRN 24/2012; TUR 7/2013; TUR 4/2013), SAT 1 (TAN 50/2012) and SAT2 (TAN 14/2012; BOT 15/2012) were used to evaluate the direct RT-LAMP-LFD assay. These epithelial suspensions were diluted 1 in 5 in nuclease free water prior to running 5 µl of each epithelial suspension (in duplicate) on the RT-LAMP-LFD assay.

Air samples used in this study were archival samples collected with portable air sampling devices during a previous animal study, the virus being of serotype Asia-1 [35].

### Preparation of RNA standards

Synthetic viral RNA was generated from plasmid pT73S containing full-length FMDV by *in vitro* transcription using a commercially available T7 RNA polymerase kit (Ambion, UK) as described by [36]. This plasmid contained the target sequences of both the one step real-time RT-PCR assay used in this study [15] as well as the region amplified by the RT-LAMP assay [24]. The resultant viral RNA was resuspended in DEPC treated water and quantified at A_260_ using a NanoDrop ND-1000 spectrophotometer. The RNA copy number concentration was calculated and adjusted to 10^9^ copies/µl.

### RNA extraction

Unless otherwise stated, total RNA was extracted by an automated procedure on a MagNA Pure LC using the total nucleic acid kit reagents following manufacturer’s guidelines.

### Reverse transcription quantitative PCR (RT-qPCR)

The RT-PCR assay used was a one-step assay that amplifies a 107 nucleotide fragment within the highly conserved 3D region of the FMDV genome, as previously described [15]. All samples assayed using this method were tested in triplicate.

### Reverse transcription LAMP (RT-LAMP)

The RT-LAMP assay used was as previously described [24], with the following modifications. The primer sequences were identical to those reported by Dukes et al [24], and given in Table S1, targeting the highly conserved RNA polymerase region of the FMDV genome (3D). The total reaction mixture was 25 µl, consisting of the following components: 2.5 µl of 10x Thermopol buffer (New England Biolabs), 1 µl of a forward internal primer (FIP)/Backward internal primer (BIP) stock mix (50 µM of each FIP/and BIP), 1 µl of an F3/B3 stock mix (5 µM each), 1 µl of each of an F Loop/B Loop stock mix (25 uM each), 0.5 µl of dNTP stock mixture (10 mM of each), 0.5 µl of MgSO_4_ (stock is 100 mM), 5 µl of Betaine (5 M stock solution), 2.2 µl of enzyme mix (*Bst* DNA Polymerase (New England Biolabs) 8 u/µl mixed with AMV Reverse Transcriptase (Promega) 10 u/µl in a volumetric ratio of 100∶1), 5 µl of a PicroGreen dye mix (molecular probes) 1.3 µl of nuclease free H_2_O and 5 µl of template RNA (or epithelial suspension). Samples were tested in triplicate. RT-LAMP reactions were run on a Stratagene Mx3005p PCR machine (Agilent Technologies) and visualised using fluorescence generated by PicoGreen intercalation and agarose gel electrophoresis imaging (AGE). Raw fluorescence was collected at one minute intervals for 60 minutes at a given incubation temperature. Exponential increase in fluorescence (δR) indicated a positive RT-LAMP reaction, irrespective of time of onset during the 60 minute incubation period [24]. The time elapsed at which point this fluorescence was detected was noted at the time to positivity (Tp).

### Reverse transcription LAMP combined with lateral flow detection (RT-LAMP-LFD)

Additional modifications which were made to the RT-LAMP assay to enable detection of the product with an LFD consisted of labelling the FIP and BIP at the 5′ terminus with fluorescein (Flc) and biotin (Btn), respectively. The subsequent reaction was referred to as RT-LAMP-LFD to distinguish it from the RT-LAMP reaction containing no labelled IP’s. RT-LAMP-LFD reactions were run using either a heat block, gradient heat block (for determining optimum temperature range 55–75°C) or using a water bath set at 60°C for one hour.

RT-LAMP-LFD reactions were visualized using agarose gel electrophoresis in combination with an immunochromatographic LFD (Forsite Diagnostics, York, UK). This device had been used in a previous study to detect LAMP products from an ASFV LAMP assay, which also incorporated biotin and fluorescein labelled primers [30]. After the 60 minute incubation step, 2 µl of the resultant RT-LAMP-LFD reaction product was added to 200 µl of LFD-Buffer C (Forsite diagnostics, York, UK) mixed well, and 75 µl of this mix was applied to the loading window of the LFD device. The mix was wicked along the device to the test line and control line. A positive result was indicated by the presence of two lines (test line and control line, respectively), while a negative result only generated a single band (control line). If no lines appeared on the LFD then it meant the test was invalid and must be run again with a new device.

## Supporting Information

Table S1 **Configuration of the oligonucleotide primers used for the LAMP-LFD amplification, includiong biotinylation [Btn] and flouresceination [Flc] sites.**
(DOCX)
