# Supplementary material for: Preliminary Validation of Direct Detection of Foot-And-Mouth Disease Virus within Clinical Samples Using Reverse Transcription Loop-Mediated Isothermal Amplification Coupled with a Simple Lateral Flow Device for Detection
Source: PLoS One. 2014 Aug 28;9(8):e105630. doi: 10.1371/journal.pone.0105630 (PMC4148330; doi:10.1371/journal.pone.0105630)
Supplement: Table S1 — Configuration of the oligonucleotide primers used for the LAMP-LFD amplification, includiong biotinylation [Btn] and flouresceination [Flc] sites. (DOCX) [file pone.0105630.s001.docx]

| Primer | Primer | Length | Sequence (5'-3') |
| --- | --- | --- | --- |
| abbreviation | name |  |  |
| F3 | Forward outer | 20-mer | CAT GGA CTA TGG AAC TGG GT |
| B3 | Reverse outer | 17-mer | GGC CCT GGA AAG GCT CA |
| FIP | Forward internal | 45-mer | [Btn] - CAC GGC GTG CAA AGG AGA GGA TTT TAC AAA CCT GTG ATG GCT |
| BIP | Backward internal | 44-mer | [Flc] - GGA GAA GTT GAT CTC CGT GGC ATT TTA AGA GAC GCC GGT ACT CG |
| FLoop | Forward loop | 18-mer | TAG CCT CGA GGG TCT TCG |
| BLoop | Reverse loop | 18-mer | GGA CTC GCC GTC CAC TCT |

**Supplementary table 1**
